# Discrepancy between performance and feedback affects mathematics student teachers’ self-efficacy but not their self-assessment accuracy

**DOI:** 10.3389/fpsyg.2024.1391093

**Published:** 2025-01-07

**Authors:** Helen M. Ernst, Anja Prinz-Weiß, Jörg Wittwer, Thamar Voss

**Affiliations:** ^1^Department of Educational Science, University of Freiburg, Freiburg, Germany; ^2^Department of Psychology, University of Education Karlsruhe, Karlsruhe, Germany

**Keywords:** self-assessment, self-efficacy, feedback, SRL, student teachers, metacognitive monitoring

## Abstract

Although feedback is of high importance for the professional development of student teachers, the impact of (inadequate) feedback on their self-regulated learning is still unclear. In two studies with mathematics student teachers, we investigated how discrepancies between performance and feedback affected two important aspects of self-regulated learning—self-efficacy and self-assessment accuracy regarding mathematical content knowledge. In the first study, *N* = 154 student teachers studying mathematics completed a knowledge test on the Pythagorean theorem and received performance feedback that was either correct or manipulated to be more positive or more negative than actual performance. The results showed that feedback that exceeded performance resulted in higher self-efficacy than feedback that fell below performance. In contrast, self-assessment accuracy in a second test on the same content was not affected by the discrepancy between student teachers’ test performance and the feedback they received. In the second study, we used the think-aloud method with *N* = 26 participants to investigate the processes underlying the effects obtained in Study 1. We found that student teachers who had received overly positive feedback were more likely to report positive affect-related statements than participants who had received overly negative or correct feedback. At the same time, they based their self-assessments in the knowledge test more strongly on their monitoring of heuristic factors than on knowledge. The results indicate that overly positive feedback elicits positive motivational states in mathematics student teachers, but bears the risk that they neglect their knowledge as a basis for their self-assessments.

## Introduction

Teacher education puts several demands on student teachers: they not only have to understand the content and didactics of their subject, but also have to monitor their own understanding in order to be able to regulate their learning and to successfully implement their knowledge in the classroom at a later point. For example, student teachers who are taught on the Pythagorean theorem need to integrate and evaluate their own knowledge and teaching methods, and adapt their lesson plans and further learning accordingly. Self-regulation of learning (SRL) is enabled in different phases of the learning process. In his cyclical theory, Zimmerman (2002) subdivides SRL in three phases: performance, self-reflection, and forethought. The performance phase is composed of self-control (e.g., focusing one’s attention on the task) and self-observation. The reflection phase consists of self-judgments that result from self-evaluations and that provoke self-reaction, such as adaptive learning behavior. The forethought phase is characterized by task analysis processes and self-motivational beliefs. Self-efficacy (i.e., the belief in one’s ability to perform a task successfully) has been identified as a key motivational construct and has been shown to be related to other motivational outcomes variables such as goal setting, effort and persistence (see Schunk and DiBenedetto, 2021).

Crucially, both the accuracy of these self-judgments in the reflection phase (i.e., self-assessments) and the degree of self-efficacy in the forethought phase have been shown to contribute to SRL (e.g., Bembenutty, 2016; Bjork et al., 2013; Lee et al., 2014; Pintrich and De Groot, 1990). Furthermore, both aspects of self-regulated learning can be improved by providing learners with feedback (Eksi, 2012; Mireles-Rios and Becchio, 2018; Sitzmann et al., 2010). On the downside, low-quality (i.e., inaccurate or biased) feedback might result in inadequate self-efficacy and inaccurate self-assessments, thus hindering adaptive SRL.

Student teachers are not regularly provided with systematic feedback about their performance, and the quality of the feedback they receive depends on the person who provides it and its intended purpose (Hudson, 2014). Therefore, it can be biased and incorrect. In two experimental studies, we examined whether correct versus false performance feedback differentially affected mathematics student teachers’ self-efficacy and self-assessment accuracy regarding their mathematical content knowledge. In the area of mathematics, good content knowledge is related to better final university degrees (Kunter and Klusmann, 2010) and facilitates learning in other knowledge domains, such as pedagogical content knowledge (Capraro et al., 2005). Most importantly, mathematical content knowledge is highly relevant for successful teaching (Blömeke et al., 2022). To measure mathematical content knowledge, objective knowledge tests provide clear scoring criteria (e.g., Backfisch et al., 2020; Krauss et al., 2008) and therefore also facilitate the examination of self-assessment accuracy through metacognitive measures. In the first study, we used a self-efficacy questionnaire and metacognitive judgments to shed light on the relationship between feedback-performance discrepancy and self-efficacy on the one hand and self-assessment accuracy on the other hand. In the second study, we relied on the same paradigm, but used the think-aloud method to gain further insight into the metacognitive, affective, and motivational processes that are elicited by (false) feedback and to provide a better understanding of the findings in Study 1.

### Self-assessment accuracy

Self-assessment is the product of a learner’s self-monitoring and evaluation of their learning process (see Panadero et al., 2017). Although self-assessments are a widely inherent part of self-regulated learning, it is also possible to prompt their use and to thus make them observable. One method used to examine self-assessments are confidence judgments, namely asking participants to assess the confidence in their response to a learning task (e.g., Bosch and Spinath, 2023). The accuracy of self-assessments can then be examined by a direct comparison between these judgments and an objective criterion (e.g., the confidence in one’s response and the correct response; Schraw, 2009). The terms *metacognitive accuracy* (e.g., Gier et al., 2009), *monitoring accuracy* (e.g., Nietfeld et al., 2006), *metacognitive monitoring accuracy* (e.g., Gutierrez de Blume, 2022), and *calibration accuracy* (e.g., Bol and Hacker, 2012) are often used alternatively to refer to self-assessment accuracy on a task-by-person level, typically indicated by confidence judgments.

Self-assessment accuracy can be examined and interpreted differentially thanks to a number of metacognitive measures: the tendency towards over- or underconfidence, the *bias*, is the signed difference between an individual’s self-assessment and a criterion. Positive values imply overconfidence and negative values imply underconfidence. *Absolute accuracy* is the squared difference between a persons’ self-assessment and a criterion. It is further possible to examine the alignment between an individual’s self-assessments and the criterion across multiple items through their correlation, which is referred to as *relative accuracy*. Metacognitive self-assessment measures are commonly used in research on learning and memory (cf. Griffin et al., 2019; meta-analyses by Panadero et al., 2017; Prinz et al., 2020), but rarely in research on (student) teachers. Drawing from research on students, accurate self-assessment has proven to be important because it allows for effective self-regulation processes (e.g., restudying), and thus can enhance academic performance (e.g., Ghanizadeh, 2017; Yan et al., 2023). Therefore, mathematics student teachers should be motivated to optimize their self-assessment accuracy to help them succeed in their studies.

### The self-efficacy mechanism

Self-efficacy—the belief in one’s capability to achieve (Voica et al., 2020)—has a strong motivational impact that enables people to overcome failures and set new personal goals (e.g., Jerusalem and Schwarzer, 1992). Bandura (1977) identified self-efficacy as a central mechanism in self-regulation and argued that self-efficacy influences how self-assessment and cognitive processing on performance operate (Bandura, 1991). His framework has empirically proven to be relevant for teacher and student outcomes (for recent meta-analyses, see Aloe et al., 2014; see also Kim and Seo, 2018). Teacher self-efficacy is positively associated with teaching performance (meta-analysis by Klassen and Tze, 2014) and job satisfaction (Dicke et al., 2015). The relationship between self-efficacy and self-regulatory processes is bidirectional: self-efficacy influences SRL, but a person’s learning experiences also shape their self-efficacy (Sitzmann and Yeo, 2013; Tolli and Schmidt, 2008).

### Empirical controversy on self-efficacy and self-assessment accuracy

While multiple studies have found a positive relationship between self-efficacy, self-regulation, and academic achievement (Bembenutty, 2016; Lee et al., 2014; Pintrich and De Groot, 1990), self-efficacy beliefs can also hinder accurate self-evaluation and result in biased self-assessments, for example, when self-efficacy beliefs are more available or salient than objective information (Ehrlinger and Dunning, 2003; Koriat, 2007). Moores and Chang (2009) identified self-efficacy as an overconfidence booster. Although self-efficacy was positively related to performance, participants performed worse when their self-efficacy had previously exceeded their performance. In line with those findings, recent studies have shown that higher self-efficacy is associated with greater overconfidence in student teachers (Ernst et al., 2023; Thomson and Nietfeld, 2017). Therefore, treatments that are designed to affect self-assessment accuracy might also affect self-efficacy, and vice versa.

### Performance feedback

Winne and Butler (1994) defined feedback as “information with which a learner can confirm, add to, overwrite, tune, or restructure information in memory, whether that information is domain knowledge, metacognitive knowledge, beliefs about self and tasks, or cognitive tactics and strategies” (p. 5740). Accordingly, feedback can affect all aspects of self-regulation, including self-assessments and self-efficacy (Panadero and Lipnevich, 2022). Feedback has proven to be an effective tool for the improvement of self-assessment accuracy. In their meta-analysis, Sitzmann et al. (2010) found that more accurate self-assessments of learning were positively related to cognitive learning outcomes (i.e., knowledge test scores) in courses that included external feedback compared to courses that did not.

There are different types and functions of feedback (for an overview, see Mory, 2004). Narciss (2004) differentiates between *outcome-related* and *elaborated feedback*. While outcome-related feedback provides information such as knowledge of performance (e.g., “5 of 7 answers were correct”), elaborated feedback provides knowledge of the correct response and additional information. In research, outcome-related feedback is often used to test the effect of feedback above no feedback or the effect of quantifiable differences in feedback characteristics such as valence and timing (Lechermeier and Fassnacht, 2018). Elaborated feedback is mostly examined in comparison to outcome-related feedback alone (e.g., Chase and Houmanfar, 2009), or is used to examine and compare different forms of elaborated feedback (e.g., Shute, 2008).

As Lechermeier and Fassnacht (2018) point out, the effect of outcome-related feedback often differs depending on its valence. Focusing on self-assessment accuracy, Eberlein et al.’s (2011) findings indicate that positive feedback on strong performance leads to greater improvements in self-assessment accuracy than negative feedback on weak performance. An effect of feedback valence has also been found regarding self-efficacy: Ryan and Deci (2000) concluded from an extensive review that feedback which is either negative or uninformative can have negative effects such as reducing self-efficacy. Accordingly, performance feedback, although being an established intervention tool, does not always elicit positive effects on self-regulatory mechanisms.

Not only correct, but also false feedback can affect self-regulatory processes. Anderson et al. (2012) found that overly positive feedback leads to overconfident self-assessments. Effects of false feedback have also been examined for self-efficacy. Overall, the results from studies in different domains (Chan and Lam, 2010; Dahling and Ruppel, 2016; Escarti and Guzman, 1999; Tolli and Schmidt, 2008; Vancouver et al., 2002, 2014) indicate that self-efficacy is increased after receiving false positive feedback and decreased after receiving false negative feedback. Overall, the reported evidence suggests that false feedback is of ambiguous value for SRL due to its seemingly antithetical effects on self-regulatory processes.

## Study 1

The findings reported above indicate that a discrepancy between feedback and performance does not only affect self-assessment accuracy, but also self-efficacy. However, these results are obtained from separate studies. It is unclear how manipulated feedback affects self-efficacy and self-assessment accuracy at the same time. Furthermore, it is an open question how this effect manifests in the population of mathematics student teachers in a content knowledge-centered performance setting. We implemented a feedback manipulation to examine the effects of the discrepancy between feedback and performance (i.e., false feedback) on mathematics student teachers’ self-efficacy and self-assessment accuracy regarding their knowledge of the Pythagorean theorem. We focused on the Pythagorean theorem because is it a central element of mathematics teacher training programs as well as of school curricula in Germany. Therefore, it bears an immediate relevance to mathematics student teachers. We expected the valence of the feedback-performance discrepancy to affect self-assessment accuracy and self-efficacy:

1) *Self-efficacy hypothesis*: The discrepancy between feedback and performance is related to task-related self-efficacy. Specifically, the more the feedback positively (vs. negatively) deviates from performance, the more self-efficacy increases (vs. decreases). A smaller discrepancy between feedback and performance leads to less systematically inflated (or deflated) self-efficacy.
2) *Self-assessment-accuracy hypothesis*: The discrepancy between feedback and performance is related to self-assessment accuracy. Specifically, the more the feedback positively (vs. negatively) deviates from performance, the greater the overconfidence (vs. underconfidence). A smaller discrepancy between feedback and performance leads to more accurate self-assessments.

We were further interested in the effects of feedback-performance discrepancy beyond the specific knowledge task. We expected the valence of the discrepancy to have an effect on the interest in correcting one’s understanding by engaging in restudy activities:

3) *Further-knowledge hypothesis*: The discrepancy between feedback and performance is related to the interest in restudying test items: specifically, the more the feedback positively (vs. negatively) deviates from performance, the lower (vs. higher) the interest in restudying.

## Method

### Sample

A total of *N* = 175 of student teachers in mathematics completed an online study which was implemented in the survey tool LimeSurvey. We excluded 21 participants because they reported that they either took notes or used online resources during test-taking. Although nine participants suggested that the effects of positive/negative feedback had been examined, none of the participants provided full knowledge of the hypotheses and therefore we did not exclude them from the analysis. Of the remaining *N* = 154 participants, 144 were in a secondary track program and 10 were in a different school track program. Participants were on average 22.56 (*SD* = 3.43) years old, 89 were female, 64 were male and one person did not indicate their gender. A power analysis conducted in G*Power (Faul et al., 2007) showed that the sample size was sufficient to detect a significant increase in *R*^2^ for a model including the feedback manipulation compared with a model that does not incorporate the feedback manipulation, with a medium effect of *f*^2^ = 0.15, a power of 0.80, and an alpha error probability of 0.05 in a stepwise multiple regression design.

### Design

**Figure 1:**
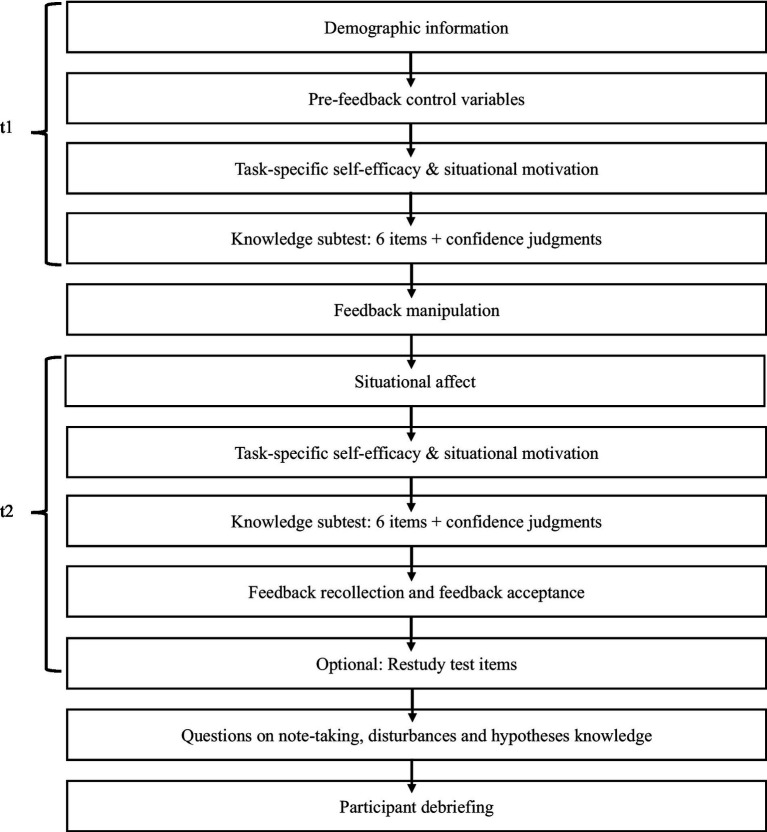
Procedure of Study 1.

The study had an experimental design and consisted of two phases (referred to as t1 and t2) with two knowledge subtests of six items, respectively. Performance feedback was provided after the first subtest. We manipulated the provided feedback in relation to participants’ subtest score to attain a positive, negative, or no discrepancy between feedback and performance. The feedback-performance discrepancy ranged from −3 (feedback = score −3) to +3 (feedback = score +3).^1^ The interval included correct performance feedback. Overall, participants could not score above six and below zero points at each subtest. Self-efficacy and self-assessment accuracy were assessed at t1 and t2, respectively. Figure 1 provides an overview of the study procedure and the used measures.

### Measures

#### Dependent variables

##### Self-assessment accuracy

Self-assessment accuracy was computed from participants’ content knowledge of the Pythagorean Theorem and their response confidence judgments of their test performance for t1 and t2, respectively. We assessed content knowledge via 12 multiple-choice items (adapted from Backfisch et al., 2020) that covered advanced knowledge on the Pythagorean theorem. An example for an item can be found in Appendix C. Based on the student performance data from Backfisch et al. (2020), we created two subtests of six multiple-choice items, respectively. Each item provided four answers of which either one or two were correct. Participants’ performance on each item was scored as either 0 (*incorrect*) or 1 (*correct*) if all correct answers were selected. Performance was measured as the percent of items solved correctly at t1 and t2, respectively.^2^ The 12 test items provided a Cronbach’s *α* of 0.66. After completing each test item, participants indicated their self-assessments as their confidence in having solved the item correctly on a 5-point scale ranging from 0 (*very unconfident*) to 4 (*very confident*). For further analyses, the scores were divided by four to reflect confidence as a relative score between 0 and 1.

Self-assessment accuracy at t1 and t2 was computed via three indices for each participant, respectively (e.g., Mengelkamp and Bannert, 2010; Schraw, 2009): (1) *Absolute accuracy* was computed as the mean of the squared absolute differences between each confidence judgment and the respective item score. Values range between 0 and 1 with smaller difference scores indicating higher accuracy. Due to the use of the square values, small deviations between confidence judgements and item scores carry less weight on the total absolute accuracy index than strong deviations; (2) *bias* is the mean of the signed differences between each confidence judgment and the respective item score with values ranging from +1 to −1. A positive bias score indicates overconfidence, whereas a negative bias score reflects underconfidence. Hence, absolute accuracy indicates the magnitude whereas bias indicates the direction of inaccurate self-assessments; (3) *relative accuracy* denotes the intraindividual correlation between the confidence judgments and item scores. This measure reflects the extent to which participants accurately distinguish items they solved correctly from items they solved incorrectly. A correlation coefficient of +1 indicates perfect relative accuracy, whereas a coefficient of −1 indicates that a participant even provided higher confidence judgments on incorrectly solved items and vice versa.

##### Task-specific self-efficacy

Test-related self-efficacy was measured as the confidence in solving a number of items in each subtest correctly. This measure was adapted from Bandura (2006). At t1 and t2, before taking each of the two subtests, participants were asked to indicate how confident they were to solve one to six out of six items (e.g., “I can solve three out of six items”), resulting in six questions for each subtest. The 5-point scale ranged from 0 (*very unconfident*) 4 (to *very confident*). Task-specific self-efficacy was then computed as the mean pre-test confidence score across the six items of each subtest, respectively. Cronbach’s *α* of the scale was 0.89 at t1 and 0.92 at t2.

##### Situational motivation

Because motivation and self-efficacy are both conceptually aligned and strongly correlated with each other, the effects of feedback are often examined for both variables and have been shown to be similar (see meta-analysis by Wisniewski et al., 2020). Task-specific situational motivation was therefore assessed as a convergent criterion variable to self-efficacy before each subtest. Hence, we adapted eight items from the German Questionnaire on Current Motivation (FAM; Rheinberg et al., 2001). A total of eight items (e.g., “When I think of the task, I am somewhat worried.”) were rated on a 5-point scale ranging from 0 (*does not apply at all*) to 4 (*fully applies*). A total mean score was computed for t1 and t2, respectively. Cronbach’s *α* of the scale was 0.70 at t1 and 0.77 at t2.

##### Intention to restudy

At the end of the study, participants could choose either to receive the test items with the correct solutions or to finish the study without receiving any further information. The choice was coded dichotomously with 0 (*no intention to study*) and 1 (*intention to restudy*).

#### Manipulation checks

##### Affect

As positive feedback increases positive feelings and negative feedback increases negative feelings (e.g., Belschak and Den Hartog, 2009), situational affect constitutes a reliable indicator of whether feedback was noticed and has actually produced arousal (Kluger et al., 1994). Therefore, we assessed participants’ affect after receiving performance feedback as a manipulation check. Participants indicated their positive and negative situational affect on the German version of the Positive and Negative Affective Schedule (PANAS; Watson et al., 1988; adapted by Krohne et al., 1996; provided by Janke and Glöckner-Rist, 2012). The questionnaire consisted of 10 adjectives indicating positive affect (e.g., “proud”) and 10 adjectives indicating negative affect (e.g., “guilty”), which were rated on a 5-point-scale ranging from 0 (*not at all*) to 4 (*very*). A mean score was computed for the positive and negative affect scale, respectively. Cronbach’s *α* was 0.86 for the positive and of 0.88 for the negative affect scale.

##### Feedback acceptance

We adapted four items from Nease et al. (1999) to assess the acceptance of the feedback (e.g., “The feedback I received was an accurate evaluation of my performance”). Participants indicated their answers on a 5-point-scale ranging from 0 (*does not apply at all*) to 4 (*fully applies*). We recoded the two inverted items and computed a mean scale score.

### Procedure

The study procedure (Figure 1) was approved by the University of Freiburg Ethics Committee (Registration No. 20-1167) and preregistered in an open access registry.^3^ Before starting the online study, participants were informed about the procedure and about data and privacy protection guidelines. They were offered eight Euros for their participation and were informed that the study would give them a chance to test their knowledge on the Pythagorean theorem. They gave their consent to participate and were then asked to provide demographic information, namely, their gender, age, study track, subject combination, semester, and university.^4^ Afterwards, the participants were informed that they would subsequently work on 12 items in two subtests to test their knowledge on the Pythagorean theorem. They were also given the information that either one or two answers could be correct in every multiple-choice item and that they would receive performance feedback after having finished the first six items. They then indicated their task motivation and self-efficacy for the first subtest and started working on the test. Each test item was presented on an individual page. After answering the item, participants indicated how confident they were in having solved it correctly.

After completion of the six items and confidence judgments, participants received performance feedback (e.g., “You have solved 3 of 6 items correctly”). Directly after receiving the feedback, participants were asked to indicate their situational affect. They then worked on the second subtest, thereby following the same procedure as before: they indicated their motivation and self-efficacy, provided answers to the items, and made confidence judgments.

Having finished the test, the participants were asked to indicate their performance in the first subtest and filled out the feedback acceptance questionnaire. They were then given the choice to either gain insight into the correct item solutions or to immediately finish the study. Finally, they were encouraged to report any disturbances during the study, note-taking activities, and their assumptions regarding the aim of the study. It was emphasized that the data quality relied on their honesty in this section and that their answers would not affect the allowance that they would receive for participating. Finally, they were informed about the feedback manipulation and their actual performance in both subtests.

### Plan of analyses

Before conducting the main hypothesis tests, we performed preliminary analyses: first, we examined changes in the main variables from t1 to t2. We then conducted manipulation checks by examining the influence of the feedback-performance-discrepancy manipulation on positive and negative affect as well as feedback acceptance. Finally, we used stepwise regressions to test the hypothesized effects of feedback-performance discrepancy on self-efficacy, bias, absolute and relative accuracy. A binary logistic regression design was applied to examine the effect of feedback-performance discrepancy on the interest in restudying the test items.

## Results

### Overall effects across time

In a first step, we examined the distributions of the variables that were assessed at measurements t1 and t2, namely performance, self-efficacy, situational motivation and the self-assessment accuracy measures. We further tested for differences between the two measurement points using *t*-tests. Table 1 provides an overview of the mean values and standard deviations of the variables at t1 and t2.

**Table 1:** Means and standard deviations of performance, self-efficacy, situational motivation, bias, absolute accuracy, and relative accuracy at t1 and t2.

Variable	t1		t2	
	*M*	*SD*	*M*	*SD*
Performance (% correct)	48.48	23.68	37.53	25.70
Task-specific self-efficacy	2.63	0.71	1.93	0.96
Situational motivation	2.93	0.48	2.54	0.64
Self-assessment accuracy				
Bias	0.00	0.20	0.06	0.25
Absolute accuracy	0.19	0.11	0.23	0.13
Relative accuracy	0.42	0.30	0.26	0.38

Relative accuracy was transformed to Fisher’s *z*-values for inferential analyses and re-transformed for the report.

For all six variables, there was a significant difference between t1 and t2 (all *p* < 0.05). Overall, participants were less motivated, provided lower self-efficacy judgments, and were less accurate in their judgments in the second subtest. Importantly, these results display a main effect for the full sample and do not indicate effects that could be traced back to the differential feedback.

### Manipulation checks

Before conducting our main analyses, we checked if situational affect and feedback acceptance were affected by the feedback that participants had received. We expected positive feedback-performance discrepancies to elicit stronger positive and that negative discrepancies would provoke stronger negative affect (Belschak and Den Hartog, 2009; Kluger et al., 1994). We conducted multiple regression analyses with positive and negative affect as well as feedback acceptance as dependent variables. As expected, performance-feedback discrepancy was significantly predictive of positive, *β* = 0.35, *p* < 0.001, and negative affect, *β* = −0.36, *p* < 0.001. Feedback acceptance was also affected: participants who had received overly positive feedback were significantly more accepting of this feedback than participants who had received overly negative feedback, *β* = 0.18, *p* = 0.022. This speaks for a higher acceptance of positive than negative feedback rather than a rejection of feedback. Altogether, these findings indicate that the feedback was processed by participants and affected their self-reports accordingly.

### Hypotheses tests

We hypothesized that the discrepancy between the actual performance and the feedback that participants receive would cause specific effects in self-efficacy and self-assessment accuracy: we expected stronger negative feedback-performance discrepancies to reduce self-efficacy and self-assessment accuracy and produce underconfident judgments whereas we assumed stronger positive feedback-performance discrepancies to increase self-efficacy but to reduce self-assessment accuracy and produce overconfidence. In addition, correct feedback was expected to result in more accurate (i.e., neither inflated nor deflated) self-efficacy and self-assessments.

We conducted stepwise regression analyses for task-related self-efficacy, self-assessment bias, absolute and relative accuracy, respectively, to test these hypotheses. For each stepwise regression, we included performance and the criterion measure at t1 and, in a second step, feedback-performance discrepancy, as predictors to account for the fact that feedback and performance were not fully independent (i.e., participants who performed faultlessly could not receive overly positive feedback, whereas participants whose answers were completely incorrect could not receive overly negative feedback). We used a linear model to test the effects on self-efficacy and bias. For absolute and relative accuracy, we did not expect a linear but a quadratic relationship between feedback-performance discrepancy and accuracy: higher negative and positive feedback-performance discrepancies were expected to result in less accurate self-assessments, while smaller discrepancies and correct feedback were expected to result in more accurate self-assessments. Therefore, we included feedback-performance discrepancy as a quadratic term in the regression analyses on these criterion variables.

#### Self-efficacy

**Figure 2:**
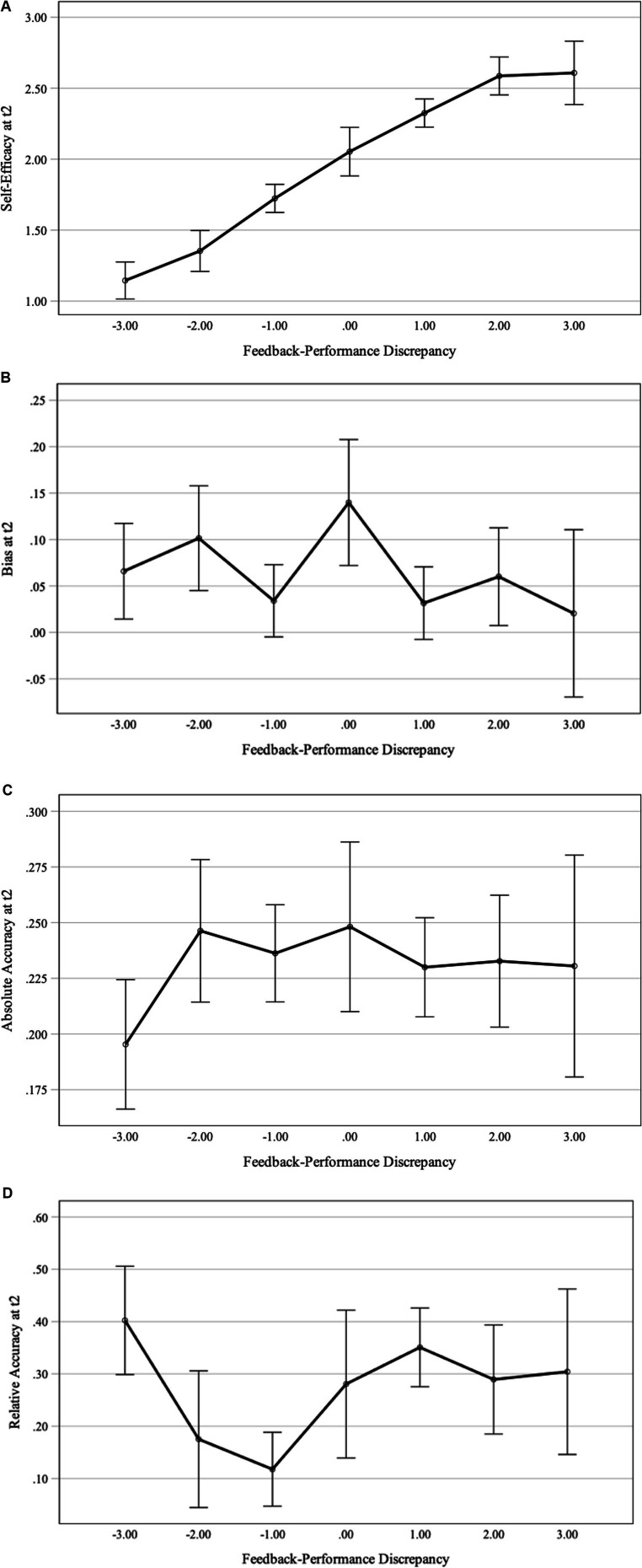
Estimated marginal means of self-efficacy **(A)**, self-assessment bias **(B)**, absolute accuracy **(C)**, and relative accuracy **(D)** at t2 by feedback-performance discrepancy. The models depict the estimated marginal means of the criterion variables at t2 with constant performance and criterion variables at t1. The error bars show standard errors.

The stepwise regression analysis showed a significant effect of feedback-performance discrepancy, *β* = 0.53, *p* < 0.001, indicating that participants who had received more favorable feedback compared to their actual performance indicated higher levels of self-efficacy before their test-taking at t2, supporting the self-efficacy hypothesis. Performance at t1, *β* = 0.47, *p* < 0.001, and task-specific self-efficacy at t1, *β* = 0.39, *p* < 0.001, were also predictive of self-efficacy at t2. But crucially, including feedback-performance discrepancy in the model significantly increased the amount of explained variance from $R_{\mathrm{step}1adj}^{2}$ = 0.37 to $R_{\mathrm{step}2adj}^{2}$ = 0.63, $R_{\Delta }^{2}$ = 0.26, *p* < 0.001. The marginal means of task-specific self-efficacy at t2 by feedback-performance discrepancy is depicted in Figure 2A. The finding was empirically supported by participants’ self-reported situational motivation, which was included as a convergent motivational measure: a regression analysis of situational motivation at t2 on feedback-performance discrepancy, *β* = 0.38, *p* < 0.001, performance, *β* = 0.40, *p* < 0.001, and situational motivation at t1, *β* = 0.38, *p* < 0.001, revealed similar results in that task-specific self-efficacy was significantly aligned to other motivational variables.

#### Self-assessment accuracy

##### Self-assessment bias

We expected a positive feedback-performance discrepancy to provoke overconfidence and, in contrast, a negative feedback-performance discrepancy to provoke underconfidence. However, the self-assessment-accuracy hypothesis was not supported by the multiple regression analysis. Bias at t1 was predictive of bias at t2, *β* = 0.39, *p* < 0.001, but performance at t1 was not, *β* = −0.09, *p* = 0.353, and neither was feedback-performance discrepancy, *β* = −0.06, *p* = 0.450. Accordingly, including feedback-performance discrepancy in the model did not significantly increase the amount of explained variance, $R_{\mathrm{step}1adj}^{2}$ = 0.17, $R_{\mathrm{step}2adj}^{2}$ = 0.17, $R_{\Delta }^{2}$ = 0.00, *p* = 0.450. Hence, participants had a tendency towards over- or underconfidence across time, but their bias at t2 could not be explained by the discrepancy between their performance at t1 and the feedback they had received (Figure 2B).

We conducted a subgroup-comparison to ensure that the lack of an effect was not a statistical artefact, namely that participants who overestimated their (weak) performance at t1 might have received mostly overly positive feedback because it was not possible for them to receive overly negative feedback to a low performance. Accordingly, participants who underestimated their (high) performance at t1 might not have had the chance to receive overly positive feedback to their high performance. We therefore specifically compared participants who over- and underestimated their performance at t1: we tested for an interaction effect of bias (under- vs. overestimation) at t1 and feedback-performance discrepancy (overly negative vs. overly positive) as dichotomized variables on bias at t2 in an ANOVA. We found a significant main effect of bias at t1, *F*(1, 122) = 21.07, *p* < 0.001, *η*^2^ = 0.15, but neither a significant main effect of feedback-performance discrepancy, *F*(1, 122) = 0.31, *p* = 0.581, nor a significant interaction effect between bias at t1 and of feedback-performance discrepancy, *F*(1, 122) = 0.05, *p* = 0.824. Namely, the direction of bias at t1 did not explain the missing effect of feedback-performance discrepancy, which supports the results of the multiple regression analysis.

##### Absolute accuracy

To test for the assumed quadratic influence of feedback-performance discrepancy on absolute accuracy, feedback-performance discrepancy was additionally included as a quadratic term in the regression. However, as for the bias index, the analysis did not support the hypothesis. Neither the linear, *β* = 0.02, *p* = 0.847, nor the squared predictor feedback-performance discrepancy, *β* = −0.08, *p* = 0.349, reached significance statistical, nor did performance at t1, *β* = −0.13, *p* = 0.112, or absolute accuracy at t1, *β* = 0.13, *p* = 0.120. The models (without vs. with feedback-performance discrepancy included) did not significantly explain any criterion variance, $R_{\mathrm{step}1adj}^{2}$ = 0.02, *p* = 0.063, $R_{\mathrm{step}2adj}^{2}$ = 0.02, $R_{\Delta }^{2}$ = 0.00, *p* = 0.578. Hence, feedback-performance discrepancy did not affect the degree of inaccuracy in the confidence judgments that participants provided (Figure 2C).

##### Relative accuracy

Just like for absolute accuracy, we included feedback-performance discrepancy as a linear and quadratic term in the regression on relative accuracy. Because each participants’ relative accuracy represented an intraindividual Pearson correlation, the values were transformed using a Fisher’s *z*-transformation for inferential analyses. Confirming the results on bias and absolute accuracy, relative accuracy was not significantly predicted through feedback-performance discrepancy (Figure 2D), neither as a linear, *β* = 0.09, *p* = 0.354, nor as a quadratic predictor, *β* = 0.14, *p* = 0.145. Only performance at t1 proved to be a significant predictor, *β* = 0.24, *p* = 0.012, but not relative accuracy at t1, *β* = 0.06, *p* = 0.507. Altogether, the analyses for the three metacognitive measures provide a homogeneous picture: the feedback manipulation did not affect the accuracy of the self-assessments that student teachers provided.

#### Interest in restudying

We had postulated in the third hypothesis that participants who received overly positive feedback would be less interested in restudying the test items and the correct answers than participants who received overly negative or correct feedback. Only 13 of 154 participants declined restudying the test material. In a binary logistic regression, the restudy choice was not significantly predicted by feedback-performance discrepancy, *B* = −0.06, *SE* = 0.17, *p* = 0.738, with an odds ratio of 0.95. Therefore, the further-knowledge hypothesis was not supported.

## Discussion

We expected the discrepancy between participants’ performance and the feedback they received after the first subtest to affect both self-efficacy and self-assessment accuracy in the second subtest, as well as the intention to restudy the test items. The data supported only the self-efficacy hypothesis: the direction of feedback was predictive of participants’ self-efficacy judgments and the result was supported by similar effects of feedback-performance discrepancy on situational motivation. This result clearly adds to empirical evidence that feedback valence has an impact on motivational variables (Fong et al., 2019; Wisniewski et al., 2020). In contrast, the feedback-performance discrepancy did not have an effect on the intention to restudy the items. Furthermore, it neither affected the accuracy of the item-specific confidence judgments nor the accuracy across items. Thus, the self-assessment-accuracy hypothesis was not supported. The findings were robust and did not change when further control variables were added to the regression models. While this finding is in contrast to Eberlein et al. (2011) and Anderson et al. (2012), it provides the implication that feedback can either harm or boost self-efficacy without inducing either over- or underconfidence. There are different theoretical explanations that could account for this phenomenon:

First, the differential effect of feedback on self-efficacy and self-assessment accuracy could mirror a postdiction-superiority effect (Pierce and Smith, 2001). Postdictions (i.e., confidence judgments that are provided after working on the item) are typically more accurate than predictions because they at least partly rely on the information provided in the test. Therefore, postdictions are typically less biased by motivational and affective states. However, the feedback information that participants in our study received after the first subtest was more accessible than the information that was presented in the test because participants did not need to extract the feedback, but it was actually presented to them. It could therefore be argued that the feedback should have affected the postdiction confidence judgments as well.

Second, the rejection of the self-assessment accuracy hypothesis could be traced back to its reliance on behavioral data. While the findings of Study 1 did not provide support for the hypothesis based on participants confidence judgments, we had not collected process data to examine the psychological reality of the mechanisms behind the behavioral variables. However, as Panadero et al. (2025) point out, self-assessment could benefit from process data to understand what learners do during their self-assessment. Therefore, we conducted a second study to challenge the assumption that the feedback-performance discrepancy could have provoked reactions on a process level without resulting in differences on the behavioral level. This exploratory study included a think-aloud instruction and focused solely on process data to identify the effect of feedback-performance discrepancy on an affective, motivational, and metacognitive level.

## Study 2

The aim of the second study was to examine differences in participants’ emotional, motivational, and metacognitive experience due to the performance feedback they receive as indicated in their think-aloud statements in the second knowledge test. Verbal protocols are typically used to gain access to processes behind psychological constructs such as reading (Magliano and Millis, 2003), problem solving (Rosenzweig et al., 2011), or SRL (Greene et al., 2011). An aim of the method is to gain verbal information without interfering with the underlying task. Therefore, participants are asked to “think aloud,” rather than being instructed to explain the reasons and processes behind their behavior (Fox et al., 2011). A growing body of research has expanded its focus on think-aloud methods to receive a better understanding of the processes behind learners’ actions: recently, Panadero et al. (2020, 2023, 2024) conducted a series of think-aloud studies to examine the self-assessment process. They found that learners differ in the strategies and criteria they use when self-assessing their performance (see Panadero et al., 2025 for an overview). While Panadero and colleagues clearly focused on metacognitive processes, think-aloud studies on feedback found evidence that affective and motivational as well as metacognitive processes were affected by the feedback participants had received (Lui and Andrade, 2022; Máñez et al., 2019). To provide a comprehensive picture, we focused on metacognitive, affective and motivational processes to identify differences that might occur as a result of the feedback but do not show in participants confidence ratings.

As this study was conducted in an exploratory manner, we proposed an open research question:

*R1*. Is there any difference regarding the content and valence of statements on a metacognitive level depending on the feedback that student teachers receive?

In Study 1, we found substantial effects of feedback-performance discrepancy on motivation (i.e., self-efficacy and situational motivation) and affect. We were interested to test if these effects would also be reflected in participants’ think-aloud statements. Therefore, we added two further research questions:

*R2*. Does overly positive (vs. overly negative) feedback have a different effect on affective statements than correct feedback?

*R3*. Does overly positive (vs. overly negative) feedback have a different effect on motivational statements than correct feedback?

## Method

### Sample

A total of *N* = 31 student teachers in mathematics completed the think-aloud study. We excluded 5 participants. Three participants figured out the manipulation during the study, one participant did not register that feedback was provided, and another participant’s audio data was of very poor quality. Therefore, the data of 26 participants (*n* = 9 for the overly negative and overly positive feedback condition respectively, *n* = 8 for the correct feedback condition) remained for analysis. Participants were on average 22.92 (*SD* = 3.20) years old, 12 were female and 14 were male.

### Measures

In this study, we focused on participants’ think-aloud statements after they had received the (manipulated) performance feedback. The control variables applied in the first study were kept in the present procedure to ensure comparability between the two studies. Participants worked on the same knowledge subtests as in the first study, but the subtests were shortened to five items each to account for the additional time that participants needed to express their thoughts while working on the test. To be able to examine pronounced effects between the feedback valences, we created three distinct categories of feedback instead of observing feedback-performance discrepancy as a continuous variable: participants were randomly assigned to one of three feedback conditions: *negative* (i.e., performance score minus 2 or a minimum of 0), *positive* (i.e., performance score plus 2 or a maximum of 5) and *correct* (i.e., performance score).

### Procedure

The study was approved by the University of Freiburg Ethics Committee (Registration No. 21-1302) and preregistered at an open access registry. The study was conducted online. Participants were informed about the procedure and aware that they would be audiotaped but that they would not be required to turn on their video cameras at any point of the study. Overall, the procedure did not differ from Study 1, apart from using the think-aloud method. The method was introduced before the assessment of situational motivation and self-efficacy took place. Participants received information about the verbalization of thoughts and worked on an example to get accustomed to thinking aloud. The recording was started and participants provided their thoughts while completing the questionnaires on self-efficacy, motivation, and affect and while providing their confidence judgements. They were not explicitly instructed to think aloud while they worked on the test items. Whenever participants were asked to report their thoughts, an instruction at the top of the page read “Please think aloud now.” Throughout the whole procedure, participants were assisted by a supervisor in an online conference tool. The supervisor reminded participants to think aloud regularly, but did not actively take part in the procedure. The recording was terminated after participants had completed the feedback acceptance questionnaire.

### Coding of think-aloud statements

Participants’ statements were transcribed from audiotapes and parsed into meaningful units. The think-aloud units were categorized based on following our research questions: metacognitive, affective, and motivational (Table 2). We also included a cognitive category based on existing literature (Kendeou et al., 2011; Prinz et al., 2019) to account for repetitions, paraphrasing, and knowledge-based inferences. The subcategories for the metacognitive processes were adapted from Prinz et al. (2019). Specifically, we distinguished three metacognitive categories based on the verbalized metacognitive monitoring: knowledge monitoring, monitoring of heuristic factors (e.g., familiarity, time spent on task), and monitoring that elicits a conceptual conflict. We added another metacognitive category that included verbal self-assessments without explicitly verbalized monitoring (examples of each category are depicted in Table 2).

**Table 2:** Coding scheme for the think-aloud units, category definitions and example quotes in Study 2.

Category	Definition	Example quote
Cognitive processes		
Repetition/paraphrasing	Repetitions of content or questions regarding the content or procedure	“I am now done with the five questions. One of five is correct”
Knowledge-based inference	Statements which imply that prior knowledge is activated or that relate the current task to prior knowledge	“But it’s definitely the case that you can use the Pythagorean theorem to check if you have a right triangle. Because otherwise, if this is not fulfilled, you do not have one”
Metacognitive processes		
Knowledge monitoring as a basis for self-assessment	Statements which show that participants are aware of the proficiency of their knowledge and use it as a basis for their self-assessments	“Another statement was about the hypotenuse being smaller. I was sure that the things that the task were about were equal in size. That’s why one is not smaller than the other. I think I can say, based on content, that I judged well”
Monitoring of heuristic factors as a basis for self-assessment	Statements that refer to (1) familiarity, (2) task complexity/time spent on the task, (3) task format/surface, or (4) other factors (e.g., luck) as a basis for self-assessments	“The task was pretty cool because I recently worked on that topic at uni. That is why I am rather confident”
Self-assessment without monitoring	Statements that do not include a further reference to monitoring processes	“And I chose one about which I am relatively confident, but unfortunately only relatively or rather. I did not choose two options about which I am relatively/or I am very sure/rather sure that they are not correct”
Conceptual change/conflict	Statements that mirror processes such as experiencing cognitive conflict or conceptual change	“It is definitely correct that my personal assessment was too high and did not match the feedback”
Affective processes		
Positive	Statements which express positive affect or its negation	“Proud, that’s not the case, not at all” (negated)
Negative	Statements which express negative affect or its negation	“Distressed, also a little bit. Probably a little bit more, substantially”
Motivational processes		
Self-efficacy	Statements which express confidence or a lack of confidence of mastering an upcoming task	“It is not that I think ‘oh yes, I will easily succeed”
Interest	Statements which express interest or a lack of interest	“I think that the next part is going to look like the first part. Therefore, my interest is slightly decreased and thus my motivation is slightly decreased”
Challenge	Statements which express effort or a lack of effort	“The feedback has definitely also affected the effort at the second part, so that is rather correct”

While the coding of metacognitive processes indicated four distinct subcategories in terms of their content, the motivational and affective statements could not unequivocally be categorized as either positive or negative because participants’ statements were closely bound to the underlying continuous scales (e.g., Rheinberg et al., 2001; Watson et al., 1988). Therefore, we adapted the underlying theoretical models for the category system: units that referred to items of the positive affect scale were subsumed under the positive affect category, while units that referred to the items of the negative affect scale were subsumed under the negative affect category. The same logic applied for the motivational categories: units that referred to self-efficacy, interest, or challenge were categorized accordingly.

One third of the statements were independently parsed and categorized by two coders. After the first round of rating, interrater reliability based on 95% unit overlap between the two coders was only moderate (Cohen’s *κ* = 0.49). Strongly diverging units were discussed and defined unanimously. The underlying subcategories were specified and the coders were trained using exemplary cases for each subcategory (see Table 2). It was also specifically defined why a unit would be included in one category, but not in another. After the second round of coding, intercoder reliability was substantially increased to Cohen’s *κ* = 0.88. The remaining divergences were solved through discussion.

## Results

### Preliminary analyses

To test for comparability of the two studies, we conducted two-sided *t*-tests of performance, self-efficacy, bias, absolute accuracy, and relative accuracy in Study 1 and 2 (for t1 and t2, respectively). Participants in Study 2 performed significantly better at t1 (*M* = 58.46% correct) than participants in Study 1 (*M* = 48.48% correct), *t*(178) = −2.00, *p* = 0.047, *d* = −0.42, and reported significantly higher self-efficacy at t2, *M*_1_ = 1.93, *M*_2_ = 2.40, *t*(178) = −2.34, *p* = 0.021, *d* = −0.50 (see Appendix D for a table of results). However, the performance effect could be explained by the deletion of item 6 in Study 2. When only the items that appeared in both studies were compared, the difference dissolved (*M*_1_ = 54.42%, *M*_2_ = 58.46%, *t*(178) = − 0.74, *p* = 0.458, *d* = −0.16), indicating that the think-aloud method did not affect performance. Neither of the comparisons between the metacognitive measures in Study 1 and Study 2 showed significant differences. Hence, thinking aloud did not systematically influence participant’s self-assessments either.

### Think-aloud data

For a quantitative analysis of the think-aloud data, we examined the frequencies of units per category at t2 by experimental condition (Table 3 for median values). We used non-parametric tests to account for the small group sizes and to account for the fact that we cannot assume normally distributed think-aloud units per category. Consistent with our research questions, we focused on metacognitive, affective, and motivational think-aloud statements. Therefore, we only instructed participants to think aloud while working on the self-report questions and providing their confidence judgments, but not while they worked on the knowledge tests items.

**Table 3:** Median (interquartile range) number of units per category at t2 by feedback condition.

Category	Negative (*n* = 9)	Correct (*n* = 8)	Positive (*n* = 9)
Total units	60.00 (43.50–66.50)	43.50 (37.25–59.50)	48.00 (43.00–58.00)
Cognitive processes			
Repetition/paraphrasing	4.00 (3.00–11.00)	5.50 (4.00–7.00)	4.00 (3.50–5.50)
Knowledge-based inference	0.00 (0.00–1.00)	0.00 (0.00–0.00)	0.00 (0.00–0.50)
Metacognitive processes			
Knowledge monitoring	2.00 (0.50–5.50)	2.00 (1.25–8.50)	2.00 (1.50–5.00)
Heuristic factors	6.00 (4.00–9.50)	4.00 (3.25–7.25)	7.00 (3.50–9.50)
Without monitoring	3.00 (0.50–5.50)	3.50 (1.00–4.75)	4.00 (1.00–4.50)
Conceptual conflict/change	3.00 (2.00–5.50)	1.00 (0.25–3.75)	2.00 (0.50–4.00)
Affective processes			
Positive (+ negations)	9.00 (8.50–9.50)	9.50 (7.50–11.00)	10.00 (9.50–10.50)
Negative (+ negations)	10.00 (9.00–10.50)	9.00 (8.25–10.00)	9.00 (8.50–9.50)
Motivational processes			
Self-efficacy	6.00 (5.00–8.50)	6.50 (3.25–7.75)	7.00 (5.00–8.00)
Interest	3.00 (2.00–3.50)	2.00 (1.00–2.00)	3.00 (2.00–3.50)
Challenge	4.00 (3.00–5.00)	3.00 (1.25–3.75)	3.00 (1.50–3.00)

Regarding the metacognitive categories, Kruskal–Wallis tests showed no significant overall effect of condition on the number of units per category (all *p* > 0.050). In a second step, we conducted an exploratory analysis to unveil effects between the metacognitive categories *knowledge monitoring* and *monitoring of heuristic factors* for each feedback condition, respectively. A Wilcoxon signed-rank test indicated that, overall, participants produced more units that relied on the monitoring of heuristic factors than of knowledge, *z*(26) = 3.15, *p* = 0.002, *r* = 0.62. However, when taking a closer look at the conditions, this difference could only be traced back to the false positive feedback condition, *z*(9) = 2.39, *p* = 0.017, *r* = 0.80, but was not found in the correct feedback group, *z*(8) = 0.94, *p* = 0.350, and negative feedback group, *z*(9) = 1.91, *p* = 0.057. Accordingly, only participants who had received overly positive feedback more often relied on heuristic factors rather than explicitly consulting knowledge for their confidence judgments.

Concerning the affective and motivational verbal categories, the feedback conditions significantly differed in the number of units in the *challenge* category, *χ*^2^(2) = 7.34, *p* = 0.026, *η*^2^ = 0.23 (Mean Rank_neg_ = 18.78, Mean Rank_cor_ = 11.38, Mean Rank_pos_ = 10.11), indicating a higher number of challenge-related statements when feedback was more negative due to the experimental condition. To gain a better understanding of the motivational and affective categories, we undertook a deeper examination of these categories. We found that the affective and motivational statements differed in whether participants explicitly referred to their performance at t1 (i.e., as indicated by the feedback they had received). In some cases, participants stated their situational affect and motivation without reference to their performance (e.g., “*Guilty*, I am somewhat surprised that *guilty* is mentioned here. I do not know why I should feel guilty […]”) or rejected any effect of performance. In other cases, they specifically connected affect and motivation to their performance (e.g., “*Proud*, rather not, three of five is not what would have met my expectations”). In Study 1, we found overly positive feedback to increase self-efficacy and positive affect and to decrease negative affect. In contrast, overly negative feedback decreased self-efficacy and positive affect and increased negative affect. Therefore, we expected differences in the references to the performance at t1 depending on whether participants had received false or correct feedback. We found a significant overall effect of feedback condition on positive affect-units, *χ*^2^(2) = 6.70, *p* = 0.035, *η*^2^ = 0.20 (Mean Rank_pos_ = 18.67, Mean Rank_cor_ = 10.44, Mean Rank_neg_ = 11.06). A significant effect of feedback condition also occurred in the challenge-related statements, *χ*^2^(2) = 10.65, *p* = 0.005. *η*^2^ = 0.38. A pairwise comparison of the conditions revealed that overly negative feedback increased the number of units that connected the expected challenge of the second test to the previously received feedback (Mean Rank_neg_ = 18.83) compared to correct (Mean Rank_cor_ = 7.88), *χ*^2^(1) = 10.96, *p*_adj_ = 0.003, *η*^2^ = 0.66, but not compared to overly positive feedback (Mean Rank_pos_ = 13.17), *χ*^2^(1) = 5.67, *p*_adj_ = 0.247, *η*^2^ = 0.29.

## Discussion

Study 2 was conducted to provide a process-oriented understanding of the results that were observed on the metacognitive, motivational, and affective level in Study 1. Overall, the number of units per category that we defined for metacognitive processes did not differ due to the feedback that participants received. Student teachers who had received false feedback did not produce more statements that reflect monitoring of knowledge, heuristic factors, or self-assessments without verbalized monitoring; neither did they engage in cognitive conflict more often than student teachers who had received correct feedback. However, we found that participants who had received overly positive feedback produced more statements in which they connected their self-assessments to heuristic factors, such as familiarity, task properties, or luck rather than connecting their self-assessment to the monitoring of their content knowledge. This finding indicates that there might be a difference in the feedback-induced metacognitive processes that could not be observed in participants’ confidence judgments in Study 1.

Unexpectedly, the student teachers only provided affective and motivational statements when they completed the self-report questionnaires, but not when they worked on the knowledge tests and provided their confidence judgments. We found that their statements were closely bound to the 5-point-Likert self-report scales that were used in the questionnaires and that it was thus not valid to categorize them dichotomously by their valence (i.e., as either positive or negative). Nonetheless, a deeper examination provided some insight in a possible effect of feedback on participants’ affective and motivational statements: we found that these statements differed in whether they did or did not include an explicit reference to the feedback that was given after the first content knowledge test. Especially participants in the positive feedback condition indicated that the feedback they had received contributed to their positive affective state. Moreover, student teachers who had received overly negative feedback indicated that their performance in the first test had an impact on feelings of challenge towards the second test. They even produced more challenge-related statements than those who had received overly positive or correct feedback. This result supports the behavioral findings from Study 1: in line with Ryan and Deci’s (2000), the overly negative feedback could have induced feelings of incompetence that were unveiled in the motivational behavioral variables and on a process level, namely reduced self-efficacy and an increased feeling of challenge.

## General discussion

The relationship between self-efficacy and self-assessment accuracy has been a topic of scientific discourse: while both self-efficacy and accurate self-assessments are mainly positively associated to SRL and performance (e.g., Bembenutty, 2016; Lee et al., 2014; Pintrich and De Groot, 1990), high levels of self-efficacy have also been connected to biased self-assessments (e.g., Thomson and Nietfeld, 2017). We conducted two consecutive experiments using different methods to examine systematic effects of feedback on self-efficacy and self-assessment accuracy on a behavioral (Study 1) and processual (Study 2) level. We introduced false (vs. correct) feedback and assessed self-efficacy and self-assessment accuracy before and after the manipulation. We did not compute the feedback in relation to participants’ self-assessments or self-efficacy predictions, but in relation to their performance. Consequently, the feedback that participants received was designed to equally affect both variables. To test our hypotheses in Study 1, we used three different metacognitive self-assessment indices: bias, absolute and relative accuracy. We included relevant pre- and post-feedback control variables and thus controlled for potential influences on the chosen criteria. Finally, we tested our hypotheses on a large sample to draw valid conclusions from our data. The results of Study 1 confirmed our hypothesis on self-efficacy, but not on self-assessment accuracy and the interest in restudying the test items. While the discrepancy between performance and feedback was predictive of student teachers’ self-efficacy as well as situational motivation and affect, it neither affected the interest to restudy the items, nor any of the metacognitive measures.

However, it is also possible that there actually was an effect of feedback on a metacognitive level that could not be observed through behavioral measures because different processes could have resulted in the same confidence judgment. Therefore, we executed a think-aloud study to gain insights on the process level behind the behavioral data of Study 1. We found that participants who had received overly positive feedback based their self-assessments more often on heuristic factors than on knowledge, whereas we did not find this effect for participants who had received correct and overly negative feedback. A look at situational affect can help to understand this effect: heuristic processing has been found to be more likely for learners in a positive affective state, while negative affect is connected to more analytic, knowledge-based processing (Forgas, 2017). In Study 1, the discrepancy between feedback and performance was predictive of positive and negative situational affect. Furthermore, participants in Study 2 indicated that their positive affect was related to the feedback they had received. Inferring from Forgas’ (2017) findings, overly positive feedback did not only increase positive feelings, but also increased the likelihood to rely on heuristic information, such as perceived task difficulty or familiarity with the subject.

These effects occurred although the participants were at least partly aware of the source of their mood, namely the feedback they had received after the first test. Accordingly, the feedback-performance discrepancy produced effects on a process level. At the same time, studies on source awareness (e.g., Gorn et al., 1993) indicate that this awareness could have prevented a mood-induced bias in participants’ confidence judgments, leading to the rejection of the self-assessment accuracy hypothesis.

### Limitations

From a methodological perspective, some limitations should be considered: our setting might have been too clinical in order to provoke a visible effect on self-assessments. Student teachers worked on a knowledge test and received automated feedback. Generally, feedback received through a computer program does not have as much impact as feedback provided by an instructor (Lipnevich and Smith, 2009). The impact might be stronger in a real-life setting when feedback is provided by a supervisor. However, this should have affected motivational processes equally and therefore is not necessarily a valid explanation for an effect on a motivational, but not on a metacognitive level. Furthermore, against common practice (see Dijkstra et al., 2008, for a review), we deliberately opted against a social comparison manipulation (e.g., feedback in relation to peers’ performance) because we were interested in the effect on participants’ intrapersonal shift in self-assessment rather than a shift due to interpersonal comparison (cf. Marsh et al., 2019).

The interest in restudying the test items with the correct solutions was very high in both studies: 92% of the participants in Study 1 and all of the participants in Study 2 chose to restudy the items. We conclude that receiving the correct test results was a strong incentive for participants, and that it thus exceeded possible motivational effects of feedback. To effectively provoke differences in the restudy choice in future studies, we would rather provide participants with restudy choices that demand effort and time investment.

Given the small sample size of Study 2, some of the underlying processes might not even have been uncovered due to a lack of statistical power. However, we addressed this issue by the use of non-parametric tests. Further, we acknowledge that our analyses were of exploratory nature and the findings should be interpreted as such. For a comprehensive quantitative analyses, we would encourage further research on the process level, especially to gain a better understanding on the metacognitive processes behind student teachers’ self-assessments.

In the present studies, we examined the effect of a singular false compared to correct outcome feedback, which we could expect to occur in real life settings in terms of biased grading and feedback processes (Malouff and Thorsteinsson, 2016). However, the choice of feedback format could have hindered an effect on self-assessment accuracy. Outcome feedback provides less guidance to self-monitoring than more elaborated, process-oriented feedback, and could therefore produce less pronounced effects in self-assessment accuracy (reviews by Brown et al., 2015; Stone, 2000). Furthermore, we cannot make assumptions on possible long-term effects of this false feedback based on our experimental design. Although longitudinal designs could provide an additional understanding of how false feedback impacts mental processes in the long run and how student teachers adjust their self-regulatory processes, the intentional use of false feedback in practice raises legitimate concerns: it would be unethical to intentionally provide student teachers with misleading feedback about their knowledge outside of a very limited experimental design that includes a thorough post-hoc debriefing. The think-aloud statements from Study 2 indicate that, although feedback does not reflect the student teachers’ actual performance, it affects the metacognitive, motivational and affective processes behind their self-assessments: especially student teachers who receive false positive feedback might rely on heuristic processing (Forgas, 2017) to assess their performance, and this could in consequence impair their SRL. Providing false feedback could further provoke other effects, such as setting inappropriate goals (Ilies and Judge, 2005), which might outweigh positive motivational effects. Instead of providing intentional false positive feedback, other approaches, such as the use of elaborated and formative (i.e., development-oriented) instead of mere outcome-related feedback should be considered to prevent a decline in self-efficacy and to enable adaptive SRL (e.g., Chan and Lam, 2010; Shute, 2008). This is further supported by Lui and Andrade (2022), who found that formative feedback overall lead to more positive than negative emotions and at the same time motivated learners to make informative meaning of the feedback they received. We would therefore encourage further research on the effect of other feedback interventions on self-assessment accuracy of teachers and prospective teachers.

We conducted this study with a specific focus on mathematics student teachers and mathematical content knowledge, with the Pythagorean theorem as a relevant topic in university and school curriculums. Prominent large-scale studies of teachers’ professional knowledge that gain their insights from the field of mathematics (e.g., COACTIV, Baumert and Kunter, 2013; TEDS-M, e.g., Blömeke and Kaiser, 2014) highlight the relevance of mathematical content knowledge in teacher education. Hence, mathematics student teachers are subject to an overwhelming amount of research on professional knowledge, as they are at a critical stage of competence development, and research findings often bare immediate implications for teacher education (see Kaiser and König, 2019). By the choice of our sample, we ensured that our findings could be interpreted in the context of existing literature and that we could produce findings relevant to research on professional competence. However, we cannot assume that our findings can directly be transferred to other populations. First, the content and format of our knowledge tests allowed for the definition of a correct answer. Carter and Dunning (2008) point out that accurate self-assessments are prone to bias when the correct answer is ill-defined or ambiguous. It is therefore unclear if the feedback could have affected self-assessment accuracy differently, had we chosen a different, less defined task (e.g., assess the quality of one’s instruction). Second, we cannot assume our feedback manipulation to elicit the same effect in a sample of in-service teachers, as knowledge and self-efficacy increase throughout the preparatory service for teaching (Schulte et al., 2008) and could be less receptive to feedback. Replications with in-service teachers and other knowledge areas could provide insights into differences and similarities and take our research a step further.

### Outlook and conclusion

Although the verbal data show some evidence that there actually is an effect of false feedback on a metacognitive level, our overall results indicate that mathematics student teachers’ motivational and affective processes are more strongly affected by feedback than metacognitive processes: taking our findings into teaching practice, we would expect student teachers who receive overly positive feedback to be happier and report higher self-efficacy, but not necessarily more inaccurate (i.e., overconfident) regarding their self-assessments than student teachers who receive overly negative or correct feedback. Praise from their lecturers or supervisors might motivate them to pursue future goals without keeping them from accurately assessing their performance. But despite the fact that we have found positive effects of false positive feedback on a motivational and affective level, we would not encourage practitioners to continuously provide student teachers with misleading feedback about their knowledge, but to find other ways to encourage them without providing them with false information.

## Data Availability

The datasets presented in this study can be found in online repositories. The names of the repository/repositories and accession number(s) can be found at: https://osf.io/dqj6x/.

## Footnotes

^1^The feedback conditions were randomly assigned. However, we needed to recode participants’ scores on one item in the first subtest. Therefore, performance at t1 and the feedback-performance discrepancy were not completely unrelated, but correlated by *r* = −0.26, *p* = 0.001.

^2^Due to an error in wording in the second knowledge test, only five of the six items were available for analysis. We calculated participants’ relative performance to preserve the comparability between test 1 (6 items) and test 2 (5 items). Overall, the test of 11 items provided a Cronbach’s *α* of 0.64.

^3^An update was added to the preregistration of Study 1 to mark necessary changes to the planned design and analyses.

^4^Overclaiming, the Big Five personality traits, prior knowledge, and self-efficacy on teaching the Pythagorean theorem, and feedback recollection were assessed as control variables. However, these variables were not the focus of our study and did not affect the interpretation of our results. A detailed description of these variables and the full analysis including these variables can be found in the Appendices A, B.
