# Correction: Discrepancy between performance and feedback affects mathematics student teachers' self-efficacy but not their self-assessment accuracy

**DOI:** 10.3389/fpsyg.2025.1687589

**Published:** 2025-09-15

**Authors:** Helen M. Ernst, Anja Prinz-Weiß, Jörg Wittwer, Thamar Voss

**Affiliations:** ^1^Department of Educational Science, University of Freiburg, Freiburg, Germany; ^2^Department of Psychology, University of Education Karlsruhe, Karlsruhe, Germany

**Keywords:** self-assessment, self-efficacy, feedback, SRL, student teachers, metacognitive monitoring

In the published article, there was an error. When the feedback conditions were introduced in the methods section of Study 2, they were interchanged. Specifically, the negative condition was labeled as *positive*, and the positive condition was labeled as *negative*. This error occurred only once. The feedback conditions were labeled correctly in the other paragraphs and the interpretation of the feedback conditions was clearly pointed out subsequently.

A correction has been made to **Study 2**, *Method, Measures*, Paragraph 1. This sentence previously stated:

“To be able to examine pronounced effects between the feedback valences, we created three distinct categories of feedback instead of observing feedback-performance discrepancy as a continuous variable: participants were randomly assigned to one of three feedback conditions: *positive* (i.e., performance score minus 2 or a minimum of 0), *negative* (i.e., performance score plus 2 or a maximum of 5) and *correct* (i.e., performance score).”

The corrected sentence appears below:

“To be able to examine pronounced effects between the feedback valences, we created three distinct categories of feedback instead of observing feedback-performance discrepancy as a continuous variable: participants were randomly assigned to one of three feedback conditions: *negative* (i.e., performance score minus 2 or a minimum of 0), *positive* (i.e., performance score plus 2 or a maximum of 5) and *correct* (i.e., performance score).”

The original article has been updated.

