# Supplementary material for: Discrepancy between performance and feedback affects mathematics student teachers’ self-efficacy but not their self-assessment accuracy
Source: Front Psychol. 2025 Jan 7;15:1391093. doi: 10.3389/fpsyg.2024.1391093 (PMC11747639; doi:10.3389/fpsyg.2024.1391093)
Supplement: Supplementary file 1 [file Data_Sheet_1.pdf]

Supplementary Material

1 Supplementary Appendix A

Description of Control Variables used in Study 1 and 2

Overclaiming. Overclaiming denotes a general tendency of a person towards self-enhancement (Ziegler et al., 2013). In past studies, overclaiming was found to be positively related to self-efficacy (Jiang & Kleitman, 2015) as well as to overconfident self-assessments (Bensch et al., 2019). We used the Vocabulary and Overclaiming Test (VOC-T; Ziegler et al., 2013) to assess a general tendency towards self-enhancement. Participants were presented with twelve words and three non-words and indicated how familiar they were with each word on a 7-point-Likert scale ranging from 1 (*I do not know the word at all*) to 7 (*I know the word very well*). The test provided an internal consistency of Cronbach's $\alpha = .78$. We computed an overclaiming tendency for each participant via signal detection analysis as the sum of relative hit rate (for words) and relative false alarm rate (for non-words) divided by two. The overclaiming tendency scores could range between 0 and 1 with higher values indicating stronger overclaiming tendencies.

Trait Personality. The Big Five personality traits openness, conscientiousness, extraversion, and neuroticism (with the exception of agreeableness) have been found to be connected to self-assessment accuracy and self-efficacy in previous studies (e.g., Dahl et al., 2010; Kim et al., 2006; Schäfer et al., 2004). We assessed trait personality using the German short scale of the Big Five Inventory (BFI-10; Rammstedt et al., 2013), which consists of ten items (i.e., two for each trait). Participants indicated their agreement on a 5-point scale ranging from 0 (*does not apply at all*) to 4 (*fully applies*). We computed a mean score for each personality trait.

Prior Knowledge. Self-reported prior knowledge on a subject has been found to be related to self-efficacy and overconfidence (Ernst et al., 2023). We used two measures to assess participants' prior knowledge on the Pythagorean theorem: (1) The participants indicated the number of attended university courses they had visited that had addressed the Pythagorean theorem; (2) the participants rated their prior knowledge on the Pythagorean theorem on a 5-point scale ranging from 0 (*no prior knowledge*) to 4 (*extensive prior knowledge*).

Self-Efficacy for Teaching the Pythagorean Theorem. We adapted the Personal Teaching Efficacy Belief scale of the Mathematics Teaching Efficacy Beliefs Instrument (MTEBI; Enochs et al., 2000; adapted from Schumacher, 2017). The scale included seven items (e.g., "I will typically be able to answer students' questions regarding the Pythagorean theorem.") and measured self-efficacy beliefs regarding the teaching of the Pythagorean Theorem. The participants indicated their answers using a 5-point scale ranging from 0 (*does not apply at all*) to 4 (*fully applies*). We computed a mean scale score for each participant. The scale provided a high reliability with Cronbach's $\alpha = .80$.

Feedback Recollection. After they had finished the second subtest, participants were asked to remember how many of the six items in the first subtest they had answered correctly. We included the difference between the recollected and the provided feedback in the main analyses to test whether incorrect feedback recollection was connected to any of the criterion variables.

2 Supplementary Appendix B

Full Model Including the Administered Covariates at t1 in Addition to Feedback-Performance Discrepancy as Predictors of the Criterion Variables at t2

Variable	Self-efficacy	Bias	Absolute accuracy	Relative accuracy [a]
	β	β	β	β
Performance at t1	.43***	-.23*	-.13	.15
Outcome variable at t1 [b]	.30***	.27*	.17*	.11
Overclaiming	.10	.15	.07	-.18
Neuroticism	-.11*	-.04	-.36***	.13
Conscientiousness	-.02	.08	.05	-.01
Extraversion	-.10	.08	.04	.01
Agreeableness	.03	-.03	-.16	.09
Openness	-.05	.08	.15	-.11
Lectures attended	-.02	.03	.00	.11
Reported knowledge	-.01	.14	-.05	.06
Self-efficacy for teaching	.15*	.06	-.03	.29**
Feedback recollection	.13*	.09	.03	.11
Feedback-performance discrepancy	.55***	-.01	-.03/.-.12	.18/.14 [c]

[a] *df* = 110. [b] Varies depending on the criterion on of the regression (e.g., self-efficacy at t1 as a predictor for self-efficacy at t2. [c] Denotes the standardized coefficient of the squared feedback-performance discrepancy.

*** $p < .05$; ** $p < .01$; * $p < .001$.

3 Supplementary Appendix C

Exemplary Tasks for the Measurement of Content Knowledge

Law of Cosines

Choose the statements that are correct for the cosine theorem:

a) The cosine theorem is a special case of the Pythagorean theorem.
b) Congruence theorems state that a triangle is completely determined by the specification of the components SAS (side-angle-side) or SSS (side-side-side). The cosine theorem allows the calculation of a fourth component, an angle (for SSS) or the third side (for SAS).
c) The cosine theorem can be proved with the help of the Pythagorean theorem
d) One formulation of the cosine theorem is: The sum of the interior angles in a triangle is 180°.

4 Supplementary Appendix D

Means, Standard Deviations, and Results of the t tests Comparing Performance (in Percent), Self-Efficacy, Bias, Absolute Accuracy, and Relative Accuracy Between Study 1 and Study 2 at t1 and t2, Respectively

	t1						t2					
Variable	Study 1		Study 2		Study 1– Study 2		Study 1		Study 2		Study 1–Study 2	
	M	*SD*	*M*	*SD*	*t*	*p*	*M*	*SD*	*M*	*SD*	*t*	*p*
Task performance	48.48	23.68	58.64	22.57	-2.00	**.047**	37.53	25.70	36.92	23.79	0.11	.910
Task-specific self-efficacy	2.63	0.71	2.72	0.54	-0.65	.516	1.93	0.96	2.40	0.88	-2.34	**.021**
Self-assessment accuracy												
Bias	0.00	0.20	0.04	0.20	-1.14	.257	0.06	0.25	0.09	0.25	-0.62	.538
Absolute accuracy	0.19	0.11	0.22	0.123	-1.08	.282	0.23	0.13	0.24	0.13	-0.24	.807
Relative accuracy	0.42	0.30	0.36	0.31	1.04 [a]	.302	0.26	0.38	0.33	0.37	-0.74 [b]	.462

Note. Relative accuracy was transformed to Fisher's *z*-values for inferential analyses and re-transformed for the report.

[a] *df* = 164. [b] *df* = 139.

References

Bensch, D., Paulhus, D. L., Stankov, L., & Ziegler, M. (2019). Teasing apart overclaiming, overconfidence, and socially desirable responding. *Assessment*, *26*(3), 351–363. https://doi.org/10.1177/1073191117700268

Dahl, M., Allwood, C. M., Rennemark, M., & Hagberg, B. (2010). The relation between personality and the realism in confidence judgements in older adults. *European Journal of Ageing*, *7*(4), 283–291. https://doi.org/10.1007/s10433-010-0164-2

Enochs, L. G., Smith, P. L., & Huinker, D. (2000). Establishing factorial validity of the mathematics teaching efficacy beliefs instrument. *School Science and Mathematics*, *100*(4), 194–202. https://doi.org/10.1111/j.1949-8594.2000.tb17256.x

Jiang, Y., & Kleitman, S. (2015). Metacognition and motivation: Links between confidence, self-protection and self-enhancement. *Learning and Individual Differences*, *37*, 222–230. https://doi.org/10.1016/j.lindif.2014.11.025

Kim, D. H., Lee, K. S., Kim, K. T., Lee, S. R., Rim, H. B., Shin, J. S., & Sohn, Y. W. (2006). The role of individual differences in confidence and response bias. *Proceedings of the Human Factors and Ergonomics Society Annual Meeting*, *50*(12), 1245–1248. https://doi.org/10.1177/154193120605001208

Rammstedt, B., Kemper, C. J., Klein, M. C., Beierlein, C., & Kovaleva, A. (2017). A short scale for assessing the Big Five dimensions of personality: 10 item Big Five Inventory (BFI-10). *Methods*, *data*, *analyses, 7*(2), 17. https://doi.org/10.12758/MDA.2013.013

Schaefer, P. S., Williams, C. C., Goodie, A. S., & Campbell, W. K. (2004). Overconfidence and the Big Five. *Journal of Research in Personality*, *38*(5), 473–480. https://doi.org/10.1016/j.jrp.2003.09.010

Schumacher, S. (2017). *Lehrerprofessionswissen im Kontext beschreibender Statistik: Entwicklung und Aufbau des Testinstruments BeSt Teacher mit ausgewählten Analysen. Bielefelder Schriften zur Didaktik der Mathematik* [Teacher professional knowledge in the context of descriptive statistics: development and structure of the BeSt teacher test instrument with selected analyses. Bielefeld writings on the didactics of mathematics]. Springer. https://doi.org/10.1007/978-3-658-17766-9

Ziegler, M., Kemper, C., & Rammstedt, B. (2013). The vocabulary and overclaiming test (VOC-T). *Journal of Individual Differences*, *34*(1), 32–40. https://doi.org/10.1027/1614-0001/a000093
